# Risk Factors for the Rupture of Middle Cerebral Artery Bifurcation Aneurysms Using CT Angiography

**DOI:** 10.1371/journal.pone.0166654

**Published:** 2016-12-15

**Authors:** Guang-xian Wang, Jiao-yan Yu, Li Wen, Lei Zhang, Ke-jie Mou, Dong Zhang

**Affiliations:** 1 Department of Radiology, Xinqiao Hospital, Third Military Medical University, Chongqing, China; 2 Department of neurosurgery, Xinqiao Hospital, Third Military Medical University, Chongqing, China; Emory University School of Medicine, UNITED STATES

## Abstract

**Background and Purpose:**

To investigate the clinical and morphological characteristics associated with risk factors for the rupture of bifurcation-type middle cerebral artery aneurysms (MCAAs).

**Methods:**

A total of 169 consecutive patients with 177 bifurcation-type MCAAs were reviewed from August 2011 to January 2016. Based on the clinical and morphologic characteristics findings, the risk factors of aneurysm rupture were assessed using statistical methods.

**Results:**

Age, cerebral atherosclerosis, no hypertension, hypertension grade 2 and coronary artery disease (CAD) were negatively correlated with aneurysm rupture. The mean diameter (*MD*) of the parent and two daughter arteries was negatively correlated with rupture. Aneurysms with irregularity, depth, width, maximum size, aspect ratio, depth-to-width ratio, bottleneck factor, and size ratio were positively correlated with rupture. The multivariate logistic regression model revealed that irregular shape (odds ratio (OR) 2.697) and aspect ratio (OR 3.723) were significantly and positively correlated with rupture, while cerebral atherosclerosis (OR 0.033), CAD (OR 0.080), and *MD* (OR 0.201) were negatively correlated with rupture. Receiver operating characteristic analysis revealed that the threshold value of the aspect ratio and *MD* were 0.96 and 2.43 mm, respectively.

**Conclusions:**

Cerebral atherosclerosis and CAD are protective factors against rupture. Morphological characteristics such as an aneurysm with an irregular shape, a high aspect ratio (>0.96) and a small *MD* (<2.43 mm) are likely better predictors of rupture.

## Introduction

The prevalence of intracranial aneurysms (IAs) is estimated to be 2%-10% of the general population [1]. The increased use of neuroimaging has led to an increase in the number of unruptured intracranial aneurysms (UIAs) found by chance. Most IAs do not rupture [2], and the use of microsurgical clipping or endovascular coiling for UIAs has risks. The decision to treat incidental UIAs is still a controversial topic in neurosurgery. Thus, the ability to predict the risk of rupture for UIAs would be of enormous clinical value. Previous studies indicated that the location was not associated with increased rupture risk after a long period of follow-up [3–6]. The International Study of Unruptured Intracranial Aneurysms (ISUIA) showed that the treatment decision regarding UIAs is based mainly on the size and location [7]. Some studies showed that the risk factors for UIAs differ by their location [8–11]. Therefore, the natural history of UIAs may be studied individually for each different location.

Middle cerebral artery aneurysms (MCAAs) are common, accounting for 18% to 40% of all IAs [12], and are responsible for up to 55% of all aneurysm-related hematomas [13]. MCAAs are divided into 3 groups according to the location of the aneurysm neck: proximal, bifurcation, and distal aneurysms [12,14], and most MCAAs belong to the bifurcation type [12,14,15]. However, only a few studies have focused on the patterns of MCAAs, and these works studied all groups and included sidewall aneurysms, which may confound the characteristics that are associated with a specific location [12,14,16]. Hence, this study was conducted to identify the relationships between personal factors and image characteristics and the rupture of bifurcation-type MCAAs.

## Materials and Methods

### Patients

This retrospective study was approved by our institutional ethics committee (Xinqiao hospital, 2011071/2016031). Signed written informed consent was obtained from all patients before the examination. A total of 22378 consecutive patients who underwent head computed tomography angiography (CTA) examinations were enrolled from August 2011 to January 2016. Out of 1176 (5.2%) patients with IAs, 220 (18.9%) presented MCAAs. Aneurysms belonging to the bifurcation type were selected, excluding proximal (n = 23) and distal aneurysms (n = 11). Subjects who had mycotic (n = 1), traumatic (n = 2), reoperated (n = 3), or fusiform aneurysms (n = 3), cases associated with arteriovenous malformations (n = 3) and poor image quality (n = 5) were also excluded. Finally, 169 patients (67 ruptured and 102 unruptured) with 177 IAs (67 ruptured and 110 unruptured) were available for analysis. Sixty-three ruptured intracranial aneurysms (RIAs) were managed with both treatment (coiling or clipping), and four patients died before surgery. Seventy UIAs were managed because of clinical symptoms (e.g., headache, dizziness), and 40 UIAs were observed with no growth or rupture signs.

The clinical data for the study were extracted from the hospital medical records by KJ Mou, who was the only person to faithfully recorded clinical data like patients with cerebral atherosclerosis or without, and blinded to the rupture or unrupture status. Cerebral atherosclerosis, coronary artery disease (CAD), and diabetes mellitus were recorded as either present or absent. Cerebral atherosclerosis was defined as diffuse atherosclerosis of the brain, luminal stenosis and small vessel occlusion, the diagnosis was made on the basis of CTA, transcranial ultrasound, or MRA. Hypertension was defined as a systolic blood pressure (BP) ≥140 mm Hg, a diastolic BP ≥90 mm Hg, or the use of antihypertensive agents. Hypertension was divided into the following 4 grades: a systolic BP <140 mm Hg or a diastolic BP<90 mm Hg as no hypertension; a systolic BP (140–159 mm Hg) or a diastolic BP (90–99 mm Hg) as grade 1; a systolic BP ≥180 mm Hg or a diastolic BP ≥110 mm Hg as grade 3; and a BP in between as grade 2. Alcohol consumption and smoking were classified as never, former and current. The history of subarachnoid hemorrhage (SAH) was defined as a history of rupture of aneurysm at another location. In cases with multiple aneurysms, the ruptured aneurysm was determined based on the location of the hemorrhage on computed tomography (CT), angiographic or operative findings.

### CTA and Image analysis

MCAAs were evaluated with CTA using a 64-slice CT machine (GE LightSpeed VCT; GE Healthcare, WI, USA). All of the images were transferred to the GE Advantix workstation (Advantage Windows 4.5) to generate 3D reconstructions and morphological measurements.

Two categorical morphological variables included the shape of the aneurysm (simple lobed or irregular shape) and the neck types. An aneurysm with lobular or daughter sacs was defined as having an irregular shape [17]. The neck types were divided according to the location of the aneurysm neck into two types: the neck located on the extension of the midline axis of the parent artery (type C) or the neck deviated from the midline axis of the parent artery (type D) [18,19].

All measurements were performed by two observers and the average value was calculated for statistical analysis. Twelve continuous morphological variables such as aneurysm depth, width, neck width, maximum size (*Dmax*), aspect ratio (*AR*), depth-to-width ratio (*DW*), bottleneck factor (*BF*), size ratio (*SR*), daughter artery ratio (*DAR*), mean diameter (*MD*) of the parent and two daughter arteries, flow angle, and lateral angle ratio (*LAR*) were examined. These variables have already been defined and are depicted clearly in the literature [1,4,10,14,16,17,18,20].

### Statistical analysis

Statistical analyses were performed using the Statistical Package for Social Sciences (SPSS, IL, USA, version 17.0). Kappa consistence test was used for the inter-observer reliability of numerical measurements. The variables were expressed as the means ± standard deviation or number of patients (%). Independent t test was used for continuous data, and chi-squared test was used for categorical data. All variables with a *P* value less than 0.2 were entered into a logistic regression model. The features that achieved univariate analysis significance (P<0.05) were further analyzed using forward multiple logistic regression to calculate the odds ratios (OR) and 95% confidence intervals (CI) for the likelihood of aneurysm rupture. Then, receiver operating characteristic (ROC) curve analysis to determine the sensitivity and specificity using the area under the curve (AUC).

## Results

### Clinical characteristics

The clinical characteristics of the 169 patients are shown in Table 1. The patients’ ages ranged from 29 to 91 years, with a mean age of 60.2±12.7 years: 58.1±13.2 years for males (range, 29–91 years), 61.6±12.2 years for females (range, 33–87 years), 64.9±11.3 years for the unruptured group (range, 33–91 years) and 53.3±11.2 years for the ruptured group (range, 29–82 years). Sixty years was chosen to dichotomize the sample because the mean patient age was 60.2 years. Based on the chi-squared test, patient age, cerebral atherosclerosis, and CAD were correlated with the risk of aneurysm rupture.

**Table 1:** Patient characteristics with ruptured and unruptured aneurysms.

Clinical data	Patient groups		*P*
	Ruptured (*n* = 67)	Unruptured (*n* = 102)	
Male	29 (43.3%)	39 (38.2%)	0.429
Age (≥60Y)†	18 (26.9%)	69 (67.6%)	<0.001
Cerebral atherosclerosis†	2 (3.0%)	51 (50.0%)	<0.001
Hypertension			
No†	38 (56.7%)	38 (37.3%)	0.013
Grade 1	4 (6.0%)	7 (6.9%)	1.000
Grade 2†	5 (7.5%)	22 (21.6%)	0.014
Grade 3	20 (29.9%)	35 (34.3%)	0.545
CAD†	1(1.5%)	12 (11.8%)	0.031
Diabetes mellitus	2 (3.0%)	10 (9.8%)	0.167
Bleeding history	4 (6.0%)	15 (14.7%)	0.131
Alcohol history			
No	51 (76.1%)	79 (77.5%)	0.841
Former	0 (0%)	1 (1.0%)	1.000
Current	16 (23.9%)	22 (21.6%)	0.725
Cigarette smoking			
No	45 (67.2%)	80 (78.4%)	0.103
Former	1 (1.5%)	1 (1.0%)	1.000
Current	21 (31.3%)	21 (20.6%)	0.114
Multiple aneurysms	15 (22.4%)	31 (30.4%)	0.253

CAD, coronary artery disease; Bleeding history, history of ruptured aneurysm in other locations.

^†^Variables showing significant difference by univariate analysis (*P*< 0.05).

### Morphologic characteristics

The level of agreement between the two observers for numerical measurements was satisfactory, with κ coefficients of 0.89 (p<0.0001). The morphological characteristics of bifurcation-type MCAAs are listed in (Table 2). Irregular shape, depth, width, *Dmax*, *AR*, *DW*, *BF*, *SR* and *MD* were associated with rupture risk.

**Table 2:** The morphological characteristics of aneurysms.

Morphologic parameters	Aneurysm groups		*P*
	Ruptured (*n* = 67)	Unruptured (*n* = 110)	
Irregular Shape†	42(62.7%)	25 (22.7%)	<0.001
Type C	43 (64.2%)	55 (50.0%)	0.066
Depth (mm)†	6.01 ± 2.95	3.91 ± 2.14	<0.001
Width (mm)†	5.71 ± 3.31	4.32 ± 2.13	0.003
Neck width (mm)	5.05 ± 2.15	4.57 ± 1.94	0.133
Maximum diameter (mm)†	7.35 ± 3.41	5.20 ± 2.60	<0.001
Aspect ratio†	1.24 ± 0.41	0.86 ± 0.34	<0.001
Depth/width ratio†	1.14 ± 0.37	0.91 ± 0.25	<0.001
Bottleneck factor†	1.14 ± 0.38	0.94 ± 0.26	<0.001
DAR	1.32 ± 0.29	1.40 ± 0.41	0.178
Mean diameter†	2.48 ± 0.36	2.72 ± 0.33	<0.001
Size ratio†	2.51 ± 1.28	1.46 ± 0.81	<0.001
Flow angle (°)	131.18 ± 29.77	137.54 ± 15.05	0.132
LAR	1.62 ± 1.11	1.60 ±1.28	0.952

DAR, daughter artery ratio; LAR, lateral angle ratio.

^†^Variables showing significant difference by univariate analysis (*P*< 0.05).

### Univariate and Multivariate analysis

**Fig 1:**
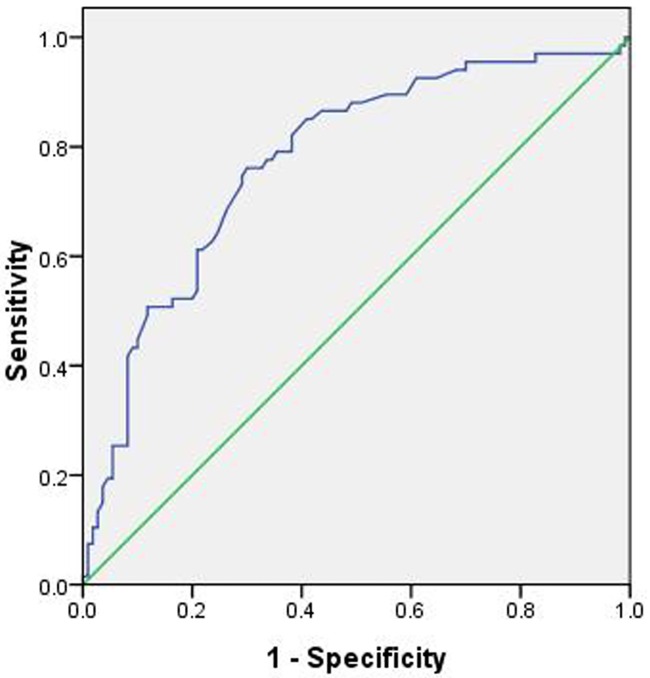
The area under the receiver operating characteristic curve for the aspect ratio is 0.774 (95% confidence interval, 0.703–0.846). The cut-off point for the aspect ratio is 0.96, the sensitivity is 76.1%, and the specificity is 70.0%.

**Fig 2:**
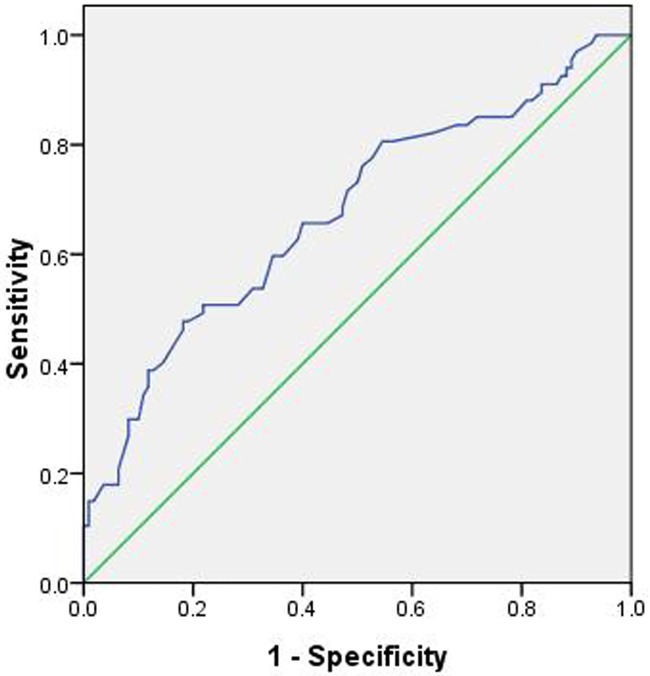
The area under the receiver operating characteristic curve for the mean diameter is 0.675 (95% confidence interval, 0.591–0.758). The cut-off point for the mean diameter is 2.43, the sensitivity is 47.8%, and the specificity is 81.8%.

Twenty-two independent variables (*P*≤0.2) were entered into a univariate logistic regression model to determine the risk factors for aneurysm rupture. Of these variables, 13 independent variables were associated with aneurysm rupture (*P*≤0.05). Then, these variables were entered into a forward conditional multiple logistic regression model (Table 3). The model showed that cerebral atherosclerosis (OR 0.033), CAD (OR 0.080), and *MD* (OR 0.201) were associated with a decreased risk of aneurysm rupture. On the contrary, aneurysms with an irregular shape (OR 2.697) and *AR* (OR 3.723) increased the risk of aneurysm rupture. The threshold values of the *AR* and *MD* were 0.96 and 2.43 mm, respectively (Figs 1 and 2), and the AUC values were 0.774 and 0.675, respectively (Table 4).

**Table 3:** Multivariate logistic regression analysis for aneurysms rupture.

Variable	Odds ratio	*P*	95% CI	β
Cerebral atherosclerosis	0.033	<0.001	0.007–0.155	–3.412
CAD	0.080	0.038	0.007–0.082	–2.520
Irregular shape	2.697	0.038	1.058–6.874	0.992
Aspect ratio	3.723	0.037	1.082–12.805	1.314
Mean diameter (mm)	0.201	0.015	0.055–0.733	–1.605

CI, Confidence intervals; CAD, coronary artery disease; β, partial regression coefficient.

**Table 4:** Area under the curve for aspect ratio and mean diameter.

Characteristics	Area	Threshold value	*P*	Sen (%)	Spe (%)	95% CI
Aspect ratio	0.774	0.96	<0.001	76.1	70.0	0.703–0.846
Mean diameter (mm)	0.675	2.43	<0.001	47.8	81.8	0.591–0.758

Sen, sensitivity, the chances of false negatives; Spe, specificity, the chance of false positives; CI, confidence intervals; Threshold value, the cut off for the aspect ratio and mean diameter.

## Discussion

Clinical and morphologic characteristics affect aneurysm rupture. Bifurcation areas of arteries are known to be vulnerable sites where the wall is weak and hemodynamic stress changes [11]. The hemodynamics of bifurcation aneurysms may be different than those of sidewall aneurysms. Additionally, different vessel diameters may have various hemodynamics. Hence, we excluded proximal and distal MCAAs to identify risk factors related to bifurcation aneurysm rupture in this study. Our results showed that cerebral atherosclerosis and CAD are protective factors against rupture of bifurcation-type MCAAs, while aneurysms with an irregular shape, a high aspect ratio and a small *MD* are risk factors for rupture.

The clinical characteristics were believed to be important factors for aneurysm rupture. However, previous studies have obtained different results. For example, patient age was reportedly positively correlated with aneurysm rupture [21], while other studies have reported that patients with RIAs were younger than those with UIAs [22,23]. The present study also had similar findings: the patients with RIAs were younger, although this factor was not significant upon multiple analysis. With increasing age, the risk of cerebral atherosclerosis is increased, and cerebral atherosclerotic or calcified walls decreased the risk of IA rupture [24]. The reason may be that cerebral atherosclerotic or calcified walls slow the flow rates entering the aneurysm and reduce wall shear stress [25]. Many studies have reported that hypertension increases the risk of aneurysm rupture [11,23,26]; however, others studies have reported that hypertension was not associated with aneurysm rupture [21,24,27]. In this study, we divided hypertension into 4 grades, and found that patients with no hypertension and grade 2 were associated with aneurysm rupture, but there was no significant difference upon multiple logistic regression. A recent study reported that CAD was a protective factor against the presence of IAs [11]. In the present study, CAD was found to be a protective factor against rupture, which is consistent with the report that patients with heart disease had a decreased risk of rupture, which may be related to restriction of strenuous physical activity [24]. In this study, risk factors such as smoking and alcohol consumption were not significantly associated with rupture. The reason may be that most patients did not have these habits, and smoking was more prominent in women than in men [28].

An aneurysm with lobular or daughter sacs was classified as having an irregular shape [17]. A previous studies reported that an irregular shape was associated with a higher risk of aneurysm rupture [6,10,14,29]. The present results also showed that irregular aneurysms are more prone to rupture, possibly because the irregular shape leads to instability of the blood flow pattern.

The ISUIA and American Heart Association/American Stroke Association showed that the treatment decision regarding UIAs is based mainly on the size and location [7,30]. A larger IA is believed to be more prone to rupture than a smaller one. The ISUIA showed a near-zero rupture risk for aneurysms less than 10 mm in diameter [22]. In fact, our study and previous studies reported that RIAs were larger than UIAs [9–11,14,16–18], but the size was not always significantly different between the ruptured and unruptured groups [16,17], which confirms the size is not a risk factor for aneurysm rupture. *AR* has been widely studied and shown to correlate with the rupture of IAs [1,4,16,18,20,30]; however, there is no consensus regarding a common threshold value in MCAAs by far. The present data showed that the threshold value of *AR* was 0.96, which is smaller than most in Caucasian populations [1].

Many previous studies reported that a larger *SR* was associated with aneurysm rupture [17,31–34]. Lin et al reported that *SR* is associated with anterior communicating artery aneurysm rupture [33] but not with MCAAs [16], but these results also showed different risk factors for different locations. In this study, the *SR* was found to be higher in the ruptured aneurysms, whereas it exhibited no relationship upon multiple analysis. *AR* and *SR* may be correlated with larger diameter MCA branches [16]. We found that *MD* was an independent predictor of rupture status in bifurcation-type MCAAs, indicating that a smaller artery is associated with a higher risk of rupture. The aneurysm arising from a small artery has a thinner wall and would experience greater wall tension [31,34].

### Limitations

The study had several limitations. Firstly, the RIAs were evaluated after rupture, the shape or size of the RIAs might have changed owing to the rupture. Although Rahman et al. [35] reported that aneurysms size don’t shrink after rupture. It is the best to prospectively investigate the IAs rupture. Secondly, UIAs may grow and evolve grossly (formation of blebs and lobes, etc.), and may rupture in the future, it is the best to dynamic observe the size and the morphological changes. But it’s very difficult to collect the serial follow-up imaging data due to the following reasons: (1) ethical issue must be taken into consideration, for example, we follow up a patient with aneurysm, and find the aneurysm become larger in size and irregular in shape, it may be rupture at any time. If we don’t treat the aneurysm, the patient maybe die due to rupture; if we do it, this case must be excluded. (2) most aneurysms do not rupture during the course of a patient’s lifetime. (3) patients with aneurysms may cause significant stress and anxiety, and want to remove the “bomb”. Lastly, although the most common site for familial IAs was the MCAA distribution [36], family history was not used in this study because the data were not recorded for many patients, especially in the elder patients.

In the future, we will need to accurately evaluate the rupture risk of aneurysms. High resolution contrast-enhanced MRI could clearly show the aneurysms wall. The rupture risk may be evaluated through measuring the MRI enhancement degree and thickness of aneurysms wall. In addition, the wall of RIAs has higher metalloproteinas-9 (MMP-9) expression than that of UIAs. We plan to synthesize the MRI probe targeted to MMP-9. Based on the enhancement degree of the aneurysms wall after injection of the probe, the rupture risk may be evaluated. All these are under our investigation.

## Conclusion

Based on the clinical records and CTA findings from patients with bifurcation-type MCAAs, we find that cerebral atherosclerosis and CAD are likely protective factors against aneurysm rupture. On the other hand, since aneurysms with an irregular shape, a high *AR* and a small *MD* are more prone to rupture; more attention should be paid to the aneurysm with these characteristics during our clinical practice.

## Supporting Information

S1 Table Patient characteristics with ruptured and unruptured aneurysms. CAD, coronary artery disease; Bleeding history, history of ruptured aneurysm in other locations. †Variables showing significant difference by univariate analysis (*P*< 0.05).
(DOCX)

S2 Table The morphological characteristics of aneurysms. DAR, daughter artery ratio; LAR, lateral angle ratio. †Variables showing significant difference by univariate analysis (*P*< 0.05).
(DOCX)

S3 Table Multivariate logistic regression analysis for aneurysms rupture. CI, Confidence intervals; CAD, coronary artery disease; β, partial regression coefficient.
(DOCX)

S4 Table Area under the curve for aspect ratio and mean diameter. Sen, sensitivity, the chances of false negatives; Spe, specificity, the chance of false positives; CI, confidence intervals; Threshold value, the cut off for the aspect ratio and mean diameter.
(DOCX)

S1 Checklist PLOS ONE Clinical Studies Checklist. (DOC)

S1 Dataset Patients and MCAAs. (XLS)
