# Supplementary material for: Risk Factors for the Rupture of Middle Cerebral Artery Bifurcation Aneurysms Using CT Angiography
Source: PLoS One. 2016 Dec 15;11(12):e0166654. doi: 10.1371/journal.pone.0166654 (PMC5157982; doi:10.1371/journal.pone.0166654)
Supplement: S1 Table — CAD, coronary artery disease; Bleeding history, history of ruptured aneurysm in other locations. †Variables showing significant difference by univariate analysis (P< 0.05). (DOCX) [file pone.0166654.s001.docx]

**Table 1.** Patient characteristics with ruptured and unruptured aneurysms

|  | **Patient groups** | |  |
| --- | --- | --- | --- |
| **Clinical data** | **Ruptured (*n* = 67)** | **Unruptured (*n* =102)** | ***P*** |
| Male | 29 (43.3%) | 39 (38.2%) | 0.429 |
| Age (≥60Y)† | 18 (26.9%) | 69 (67.6%) | <0.001 |
| Cerebral atherosclerosis† | 2 (3.0%) | 51 (50.0%) | <0.001 |
| Hypertension |  |  |  |
| No† | 38 (56.7%) | 38 (37.3%) | 0.013 |
| Grade 1 | 4 (6.0%) | 7 (6.9%) | 1.000 |
| Grade 2† | 5 (7.5%) | 22 (21.6%) | 0.014 |
| Grade 3 | 20 (29.9%) | 35 (34.3%) | 0.545 |
| CAD† | 1(1.5%) | 12 (11.8%) | 0.031 |
| Diabetes mellitus | 2 (3.0%) | 10 (9.8%) | 0.167 |
| Bleeding history | 4 (6.0%) | 15 (14.7%) | 0.131 |
| Alcohol history |  |  |  |
| No | 51 (76.1%) | 79 (77.5%) | 0.841 |
| Former | 0 (0%) | 1 (1.0%) | 1.000 |
| Current | 16 (23.9%) | 22 (21.6%) | 0.725 |
| Cigarette smoking |  |  |  |
| No | 45 (67.2%) | 80 (78.4%) | 0.103 |
| Former | 1 (1.5%) | 1 (1.0%) | 1.000 |
| Current | 21 (31.3%) | 21 (20.6%) | 0.114 |
| Multiple aneurysms | 15 (22.4%) | 31 (30.4%) | 0.253 |

CAD, coronary artery disease; Bleeding history, history of ruptured aneurysm in other locations.

†Variables showing significant difference by univariate analysis (*P*< 0.05).
