# Supplementary material for: Risk Factors for the Rupture of Middle Cerebral Artery Bifurcation Aneurysms Using CT Angiography
Source: PLoS One. 2016 Dec 15;11(12):e0166654. doi: 10.1371/journal.pone.0166654 (PMC5157982; doi:10.1371/journal.pone.0166654)
Supplement: S2 Table — DAR, daughter artery ratio; LAR, lateral angle ratio. †Variables showing significant difference by univariate analysis (P< 0.05). (DOCX) [file pone.0166654.s002.docx]

**Table 2.** The morphological characteristics of aneurysms

|  | **Aneurysm groups** | |  |
| --- | --- | --- | --- |
| **Morphologic parameters** | **Ruptured (*n* = 67)** | **Unruptured (*n* = 110)** | ***P*** |
| Irregular Shape† | 42(62.7%) | 25 (22.7%) | <0.001 |
| Type C | 43 (64.2%) | 55 (50.0%) | 0.066 |
| Depth (mm)† | 6.01 ± 2.95 | 3.91 ± 2.14 | <0.001 |
| Width (mm)† | 5.71 ± 3.31 | 4.32 ± 2.13 | 0.003 |
| Neck width (mm) | 5.05 ± 2.15 | 4.57 ± 1.94 | 0.133 |
| Maximum diameter (mm)† | 7.35 ± 3.41 | 5.20 ± 2.60 | <0.001 |
| Aspect ratio† | 1.24 ± 0.41 | 0.86 ± 0.34 | <0.001 |
| Depth/width ratio† | 1.14 ± 0.37 | 0.91 ± 0.25 | <0.001 |
| Bottleneck factor† | 1.14 ± 0.38 | 0.94 ± 0.26 | <0.001 |
| DAR | 1.32 ± 0.29 | 1.40 ± 0.41 | 0.178 |
| Mean diameter† | 2.48 ± 0.36 | 2.72 ± 0.33 | <0.001 |
| Size ratio† | 2.51 ± 1.28 | 1.46 ± 0.81 | <0.001 |
| Flow angle (°) | 131.18 ± 29.77 | 137.54 ± 15.05 | 0.132 |
| LAR | 1.62 ± 1.11 | 1.60 ±1.28 | 0.952 |

DAR, daughter artery ratio; LAR, lateral angle ratio.

†Variables showing significant difference by univariate analysis (*P*< 0.05).
