# Supplementary material for: Risk Factors for the Rupture of Middle Cerebral Artery Bifurcation Aneurysms Using CT Angiography
Source: PLoS One. 2016 Dec 15;11(12):e0166654. doi: 10.1371/journal.pone.0166654 (PMC5157982; doi:10.1371/journal.pone.0166654)
Supplement: S3 Table — CI, Confidence intervals; CAD, coronary artery disease; β, partial regression coefficient. (DOCX) [file pone.0166654.s003.docx]

**Table 3.** Multivariate logistic regression analysis for aneurysms rupture

| **Variable** | **Odds ratio** | ***P*** | **95% CI** | **β** |
| --- | --- | --- | --- | --- |
| Cerebral atherosclerosis | 0.033 | <0.001 | 0.007–0.155 | –3.412 |
| CAD | 0.080 | 0.038 | 0.007-0.082 | –2.520 |
| Irregular shape | 2.697 | 0.038 | 1.058–6.874 | 0.992 |
| Aspect ratio | 3.723 | 0.037 | 1.082–12.805 | 1.314 |
| Mean diameter (mm) | 0.201 | 0.015 | 0.055–0.733 | –1.605 |

CI, Confidence intervals; CAD, coronary artery disease; β, partial regression coefficient.
