# Supplementary material for: Risk Factors for the Rupture of Middle Cerebral Artery Bifurcation Aneurysms Using CT Angiography
Source: PLoS One. 2016 Dec 15;11(12):e0166654. doi: 10.1371/journal.pone.0166654 (PMC5157982; doi:10.1371/journal.pone.0166654)
Supplement: S4 Table — Sen, sensitivity, the chances of false negatives; Spe, specificity, the chance of false positives; CI, confidence intervals; Threshold value, the cut off for the aspect ratio and mean diameter. (DOCX) [file pone.0166654.s004.docx]

**Table 4.** Area under the curve for aspect ratio and mean diameter

| **Characteristics** | **Area** | **Threshold value** | ***P*** | **Sen (%)** | **Spe (%)** | **95% CI** |
| --- | --- | --- | --- | --- | --- | --- |
| Aspect ratio | 0.774 | 0.96 | <0.001 | 76.1 | 70.0 | 0.703–0.846 |
| Mean diameter (mm) | 0.675 | 2.43 | <0.001 | 47.8 | 81.8 | 0.591-0.758 |

Sen, sensitivity, the chances of false negatives; Spe, specificity, the chance of false positives; CI, confidence intervals; Threshold value, the cut off for the aspect ratio and mean diameter.
